# Screening for Genes Related to Meat Production Traits in Duroc × Bama Xiang Crossbred Pigs by Whole Transcriptome Sequencing

**DOI:** 10.3390/ani14162347

**Published:** 2024-08-14

**Authors:** Yupei Xu, Hui Wang, Feng Cheng, Kuirong Chen, Guofeng Lei, Zhongrong Deng, Xiaoxiao Wu, Cong Liu, Jinglei Si, Jing Liang

**Affiliations:** 1 College of Animal Science & Technology, Guangxi University, Nanning 530004, China; 15866005271@163.com (Y.X.); w1459698724@163.com (H.W.); cf52597@163.com (F.C.); 15737555093@163.com (K.C.); 15885966834@163.com (G.L.); 15213036981@163.com (Z.D.); 18579933943@163.com (X.W.); 18839630895@163.com (C.L.); 2 Guangxi State Farms Yongxin Animal Husbandry Group Co., Ltd., Nanning 530022, China

**Keywords:** meat production, pig, whole transcriptome sequencing, ceRNA regulatory network, *longissimus dorsi*

## Abstract

**Simple Summary:**

Growth and meat production traits are significant economic traits in pigs. In this study, we investigated the differences in phenotypic traits as well as the genes and pathways affecting meat production traits in individuals from Duroc × Bama Xiang F2 crossbred pigs with different growth rates. The regulatory relationships between genes and meat production traits were explored by utilizing whole transcriptome sequencing technology and ceRNA regulatory network analysis. The results of this study provide a reference for the genetic basis analysis of porcine meat production traits.

**Abstract:**

The meat production traits of pigs are influenced by the expression regulation of multiple gene types, including mRNAs, miRNAs, and lncRNAs. To study the differences in meat production traits at the transcriptional level among individuals with different growth rates, the *longissimus dorsi* samples from eight Duroc × Bama Xiang F2 crossbred pigs with a fast growth rate (high gTroup) or a slow growth rate (low group) were selected to perform whole transcriptome sequencing and ceRNA regulatory network construction. This study first analyzed the differences in physiological and biochemical indicators, muscle histological characteristics, and muscle fiber types. A total of 248 mRNAs, 25 miRNAs, and 432 lncRNAs were identified as differentially expressed by whole transcriptome sequencing. Key genes that may influence meat production traits include *MTMR14*, *PPP1R3A*, *PYGM*, *PGAM2*, *MYH1*, and *MYH7*. The ceRNA regulatory network map showed that ENSSSCG00000042061-ssc-mir-208b-*MYH7*, ENSSSCG00000042223-ssc-mir-146a-*MTMR14*, ENSSSCG00000045539-ssc-mir-9-3-*MYH1*, and ENSSSCG00000047852-ssc-mir-103-1-*PPP1R3A* may be the key factors affecting meat production traits through their regulatory relationships. This study provides valuable insights into the molecular mechanisms underlying porcine muscle development and can aid in improving meat production traits.

## 1. Introduction

Pigs are a primary source of meat for humans, accounting for about 30% of meat consumption worldwide [1]. Pork provides essential nutrients such as protein, fat, and iron [2] and has potential benefits for human disease treatment [3]. Pig production is a significant component of the animal husbandry economy, which forms the backbone of the global agricultural economy [4]. The Duroc pig, a commercial lean breed originating from the United States, is known for its thin backfat, high lean meat rate, and fast growth rates [5]. In contrast, the Bama Xiang pig, a famous breed from Bama County, Guangxi, China [6], is renowned for its excellent meat quality, with rich intramuscular fat and unsaturated fatty acid-rich meat [7]. However, Bama Xiang pigs have the disadvantage of slow growth [8]. To combine the fast growth rate of Duroc pigs with the superior meat quality of Bama Xiang pigs, crossbreeding was conducted. In this study, we measured the meat production traits and performed transcriptomic analysis of the *longissimus dorsi* to explore the genetic basis of the meat production traits in the Duroc × Bama Xiang crossbred population.

The growth and development of skeletal muscle are closely related to meat production and are the main factors affecting overall growth [9]. Pigs represent a major source of meat production worldwide and are an ideal animal model in myogenesis studies [10]. Their growth and development are complex biological processes involving the interaction of multiple functional genes and the environment [11]. Myofibers are derived from myoblasts, which undergo proliferation and fusion to form myotubes and ultimately differentiate into mature myofibers [12]. Muscle is composed primarily of muscle fibers, adipose tissue, and connective tissue, and their nature and the relative proportions of each determine the meat’s quality. Skeletal muscle quantity and quality are considered the main indicators of meat quality [13]. Deciphering the molecular mechanisms that regulate skeletal muscle development and growth is significant for both animal husbandry and biomedicine [14].

The transcriptome is the set of all RNA molecules transcribed into a cell or population of cells at a particular developmental stage, including mRNAs, rRNAs, tRNAs, and other non-coding RNAs [15,16]. The creation of transcriptome databases helps to identify the differential gene expression within different cell groups. Whole transcriptome analysis is performed with the aim of sequencing to obtain coding and non-coding RNAs and subsequently exploring the differential factors of gene expression [17]. RNA-seq is a high-throughput sequencing technology that allows the rapid identification and analysis of the vast majority of the transcriptome [18]. Many RNA-seq studies have been carried out in pigs to assess the differences found in the transcriptome of muscle, fat, liver, or hypothalamus among breeds or phenotypically extreme individuals within a breed for characters of interest [19,20]. Sequencing of the mRNA transcriptome has been extensively studied in the livestock industry, including swine and poultry, which has yielded more direct implications for analyzing gene function [21]. MiRNAs play critical roles in fat deposition and energy metabolism [22]. LncRNAs are key factors in controlling gene expression [23] and are associated with adipogenesis and lipid metabolism in pigs [24]. Meat production regulation is a complex process regulated by multiple genes involving mRNAs, miRNAs, and lncRNAs. lncRNAs, for example, are involved in the regulatory network of adipogenesis in a variety of ways to influence the content of fat deposits, while miRNAs also play an important role in adipogenesis [25]. In order to explore the functional candidate genes affecting pig meat production traits at the transcriptome level, we used transcriptome sequencing technology to screen the mRNAs, miRNAs, and lncRNAs differentially expressed in the *longissimus dorsi* of different phenotypes of crossbred pigs and to construct the ceRNA regulatory network.

## 2. Materials and Methods

### 2.1. Ethics Statement and Sample Preparation

All animal procedures were approved by the Ethics of Animal Experiments of Guangxi University (GXU2018-006).

Eight Duroc (♂) × Bama Xiang (♀) F2 generation crossbred pigs (270 ± 5 days old) were selected and raised under the same conditions. Four individuals (two males and two females) with fast and slow growth rates were divided into high- and low-growth-rate groups, respectively. The pigs were fasted for 24 h and humanely slaughtered the following day. Carotid blood was collected before slaughter, and fresh blood samples were stored at 4 °C. After slaughter, the *longissimus dorsi* muscle was collected from the same anatomical location in each pig, immediately frozen in liquid nitrogen (−196 °C), and then transferred to a −80 °C freezer for RNA extraction.

### 2.2. Determination of Carcass and Meat Phenotypic Traits and Plasma Biochemical Indicators

Electronic scales (Guilin Measuring and Grinding Tools Co., Ltd., Guilin, China) and electronic vernier calipers (Guilin Gauge and Sharpening Tools Co., Ltd., Guilin, China) were used to determine the carcass weight and backfat thickness after slaughter. A pH meter (MATTHAUS, Wissen, Germany) was used to determine the pH value 45 min and 24 h after slaughter. Meat color was scored on an OPTO-STAR colorimeter (MATTHAUS, Wissen, Germany) and meat color comparison board for meat samples 24 h after slaughter. Share force was determined 45 min after slaughter using a water bath (Shanghai Boxun Medical & Biological Instrument Co., Ltd., Shanghai, China) and a shear tester (Nanjing Ming’ao Instrument and Equipment Co., Ltd., Nanjing, China). Drip loss was determined using an electronic balance (Shanghai Sunyu Hengping Scientific Instrument Co., Ltd., Shanghai, China). The intramuscular fat content was determined by the Soxhlet extraction method using the weights before and after extraction after drying the samples in the electrically heated constant temperature blast dryer (Shanghai Boxun Medical and Biological Instrument Co., Ltd., Shanghai, China). Recordings of the fresh meat weight, drying constant weight, and moisture content of the samples were determined by the direct drying method.

Fresh blood samples were analyzed by using a PE−6800 VET Automatic Blood Cell Analyzer (Shenzhen Pukang Electronics Co., Ltd., Shenzhen, China). The plasma biochemical indicators were measured by using the JiuBang biological kit (Quanzhou Jiubang Biotechnology Co., Ltd., Quanzhou, China), including the total protein, cholesterol, triglycerides, creatinine, glucose, urea nitrogen, and were conducted in strict accordance with the instructions. The UV–visible spectrophotometer (Tecan, Zurich, Switzerland) and biochemical analyzer (BioTek, Winooski, VT, USA) were also used in the process.

### 2.3. NADH-TR Staining and HE Staining of Myofibers

Cross-sectional sections of myofibers were prepared using the LEICA CM3050S frozen microtome (Shanghai Leica Instruments Co., Ltd., Shanghai, China). Frozen sections of 10 μm were stained with the NADH myocardial xanthanase activity staining kit (Shanghai Haling Biotechnology Co., Ltd., Shanghai, China). NADH-TR staining solution (Shanghai Haling Biotechnology Co., Ltd., Shanghai, China)is reduced by oxidase in the myofibers to produce insoluble blue-melanin deposits on skeletal muscle fibers. Type I myofibers have a higher content of oxidase than type II, so type I myofibers show a dark blue-purple color after staining, and type II myofibers show a blue-purple or light blue-purple color. The different fiber types were determined by varying degrees of the color of nicotinamide adenine dinucleotide tetrazolium reductase (NADH-TR) staining by referring to the NADH myocardial xanthanase activity staining kit product manual (HL80039.2 V.A). The slide pasted with slices (Jiangsu Shitai Experimental Equipment Co., Ltd., Nanjing, China) was stained with HE, additionally. The hematoxylin staining solution (servicebio) was stained for 45 s and then rinsed slowly in tap water for 8 s. The process of rinsing in tap water was repeated after staining with an eosin staining solution (Wuhan Servicebio Biotechnology Co., Ltd., Wuhan, China) for 12 s. The slices were sealed with neutral gum and then placed in a ventilated place to air-dry. The sections were observed by fluorescence microscope, photographing under 20× magnification, and the diameter of the muscle fibers was measured by Image J software (Version 1.53q) (NIH, Bethesda, MD, USA) using muscle fiber bundles as the units. The number of muscle fiber bundles measured was at least 10, and the diameter of the muscle fibers was calculated by fitting the cross-section of the muscle fibers into a circle, and the diameter was calculated and averaged.

### 2.4. Library Preparation and RNA Sequencing

The total RNA was extracted with a Trizol reagent (Vazyme, Nanjing, China). The extracted RNA was analyzed by using the UV–visible spectrophotometer (Tecan, Zurich, Switzerland) for quality testing. Eligible RNA was reverse-transcribed using a CFX96 quantitative PCR instrument (BioTek, Winooski, VT, USA). According to the manufacturer’s instructions, the cDNA library was constructed by the kit (Illumina, San Diego, CA, USA) and then sequenced on the DNBSEQ platform. Raw data were filtered using Fast QC software (version 0.11.5) to obtain valid data for the subsequent analysis, removing reads shorter than 18 nt (excluding adapters). After removing these reads using sequencing connectors, sequencing primers, and low-quality scores, the clean reads were aligned to the porcine reference genome (*Sus scrofa* 11.1) via HISAT2 (2.2.1). Binary alignment map (BAM) files were created and sorted using SAMtools (1.14). The clean reads were aligned against the GenBank database (version 209.0) and the Rfam database to identify and remove rRNAs, scRNAs, snoRNAs, snRNAs, and tRNAs. Known miRNAs were identified by alignment with the miRbase database (Release 21). Known miRNAs were also identified based on the Mireap_v0.2 software-predicted genomic locations and hairpin structures, and new miRNA candidates were identified. The expression level of the miRNAs was quantified using the number of million markers (TPM) algorithm.

### 2.5. Differential Expression and Functional Analysis of mRNA, miRNA, and lncRNA 

The differential expression analysis of mRNAs was carried out by using the R package DESeq2 (1.30.1). The differentially expressed genes were identified with a fold-change ≥ 1 and a *p*-value < 0.05. DEGs were clustered and analyzed using the clustering analysis on the Ouyi BioCloud platform (www.oebiotech.cn./tools, accessed on 8 September 2022). All the differentially expressed genes were subjected to GO enrichment and Kyoto Encyclopedia of Genes and Genomes (KEGG) pathway analyses using the KOBAS database (http://bioinfo.org/kobas, accessed on 26 September 2022). The significantly enriched terms were filtered using a *p*-value threshold of 0.05. TargetScan 50 was used to identify the miRNA binding sites, and the predicted target genes were used as candidate target genes for the next step of the experiment. Screening for differentially expressed miRNAs was conducted using high-throughput sequencing with a fold-change ≥ 1 and a *p*-value < 0.05 and annotated using the GO terms and KEGG pathway of these most abundant miRNAs. The screening criteria of the significantly enriched terms were the same as the mRNA. The prediction of lncRNA in novel transcripts using CNCI (coding-non-coding index) analysis, CPC (coding potential calculator) analysis, CPAT, and Pfam proteins domain analysis was to determine whether they have coding potential. The differentially expressed lncRNAs were identified with a fold-change ≥ 1 and a *p*-value < 0.05, followed by GO enrichment and KEGG pathway analyses.

### 2.6. Quantitative Real-Time PCR (qRT-PCR) Validation

RNAs were extracted from the *longissimus dorsi* of hybrid pigs using the Trizol method. RNAs with satisfactory results were reverse-transcribed using a quantitative PCR instrument (CFX96) (BioTek, Winooski, VT, USA). cDNAs were used as templates, diluted 10-fold, and stored at 4 °C (Midea Co., Ltd., Foshan, China) for subsequent manipulation. The specific primer sequences for the candidate differentiation genes of mRNAs and lncRNAs and the endogenous reference GAPDH were designed by oligo7, and then primer synthesis was performed (Janus Bio, Nanning, China). miRNA fluorescence quantification was performed by using the stem-loop method using the Vazyme online software (miRNA Design V1.01) (Vazyme). miRNA quantification primers were designed and synthesized using the Vazyme online software (miRNA Design V1.01) (Vazyme). And the cDNAs of miRNA and internal reference U6 were synthesized by reverse-transcription using the miRNA 1st Strand CDNA Synthesis Kit (by stem-loop) (Vazyme). The cDNA was synthesized by reverse-transcription of miRNA and the internal reference U6, and the cDNA was synthesized by the stem-loop primer reaction. The cDNA was used as a template and diluted 10-fold for subsequent operations. The results of the real-time fluorescence quantitative PCR were compared to the analytical results of high-throughput sequencing for trend agreement. The relative expression levels of differentially expressed genes were analyzed by the 2^−ΔΔCT^ method against the *GAPDH* gene.

### 2.7. Analysis of ceRNA Regulatory Network

Differently expressed miRNAs corresponding to differentially expressed lncRNAs and differentially expressed mRNAs were predicted using MIREAP_v0.2, miRanda, and TargetScan. The expression correlation between differentially expressed miRNAs and their targets (lncRNA-miRNAs or miRNA-mRNAs) was calculated using Spearman’s rank correlation coefficient (SCC). Those with a SCC of less than −0.7 were selected as candidate lncRNA-miRNAs or miRNA-mRNAs. The expression correlation between differentially lncRNAs and miRNAs was calculated using the SCC, and those with a SCC greater than 0.9 were used as candidate ceRNAs (lncRNA-mRNAs). The hypergeometric cumulative distribution function test in R was utilized to identify the final ceRNA pairs (*p* < 0.05). Based on the results of target prediction, expression correlation, as well as the hypergeometric cumulative distribution test, the lncRNA-miRNA-mRNA networks were visualized using Cytoscape (v3.8.2) (http://www.cytoscape.org/, accessed on 26 October 2022). The NetworkAnalyzer plug-in of Cytoscape was used to calculate the connectivity. Nodes with a degree of connectivity greater than the average degree of the entire ceRNA network were identified as highly connected genes.

### 2.8. Statistical Analysis

Data were analyzed by one-way ANOVA as well as an independent samples *t*-test using IBM SPSS Statistics 25 software (IBM, Armonk, NY, USA). Levene statistics was used to judge the chi-square, and the normal distribution curve indicated that the data conformed to a normal distribution. Data are presented as the mean ± SD. A *p* < 0.05 indicates significant differences, and a *p* < 0.01 indicates highly significant differences. Data were visualized using GraphPad Prism 8 software (GraphPad, San Diego, CA, USA).

## 3. Results

### 3.1. Analysis of Carcass and Meat Phenotypic Traits and Plasma Biochemical Indicators

The differences in the slaughter and meat quality indexes of the F2 generation crossbred pigs were analyzed. It was found that the loin eye area was significantly higher in the high group than in the low group (*p* < 0.05), the backfat thickness of the thoracolumbar junction was significantly lower in the high group than in the low group (*p* < 0.05), the pH 24 h was significantly higher in the high group than in the low group (*p* < 0.05), and there were no significant differences in the other indexes (Table 1). Analysis of the routine serum biochemical indices showed that the total protein and cholesterol in crossbred pigs were significantly lower in the high group than in the low group (*p* < 0.05), and the differences in the other biochemical indices were not significant (Table 2).

### 3.2. Comparison of Muscle Histological Properties and Muscle Fiber Types

**Figure 1:**
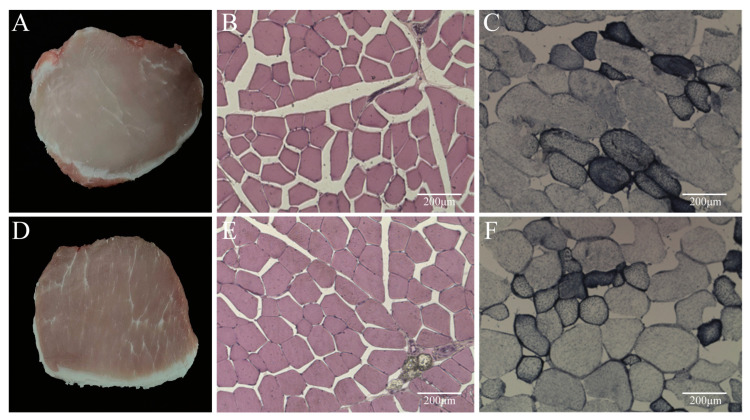
Observation of phenotype and histological characteristics of the *Longissimus Dorsi.* (**A**) Cross-section of the *longissimus dorsi* in the high group. (**B**) HE staining of the *longissimus dorsi* myofibers in the high group. (**C**) NADH-TR staining of the *longissimus dorsi* myofibers in the high group. (**D**) Cross-section of the *longissimus dorsi* in the low group. (**E**) HE staining of the *longissimus dorsi* myofibers in the low group. (**F**) NADH-TR staining of the *longissimus dorsi* myofibers in the low group.

A clear difference in the marbling contents between the high and low groups can be seen from the cross-sectional pictures of the *longissimus dorsi* (Figure 1A,D). The HE staining of frozen sections of the *longissimus dorsi* tissues of hybrid pigs, observed under a fluorescence microscope, revealed that there was no significant difference in the fiber diameters in the high and low groups (Table 3) (Figure 1B,E). The NADH-TR staining of the *longissimus dorsi* myofibers showed less blue-black in the high group, indicating fewer type I fibers. In contrast, there was relatively more blue-black in the low group, indicating more type I fibers (Table 4) (Figure 1C,F).

### 3.3. mRNA Sequencing Expression Analysis

**Figure 2:**
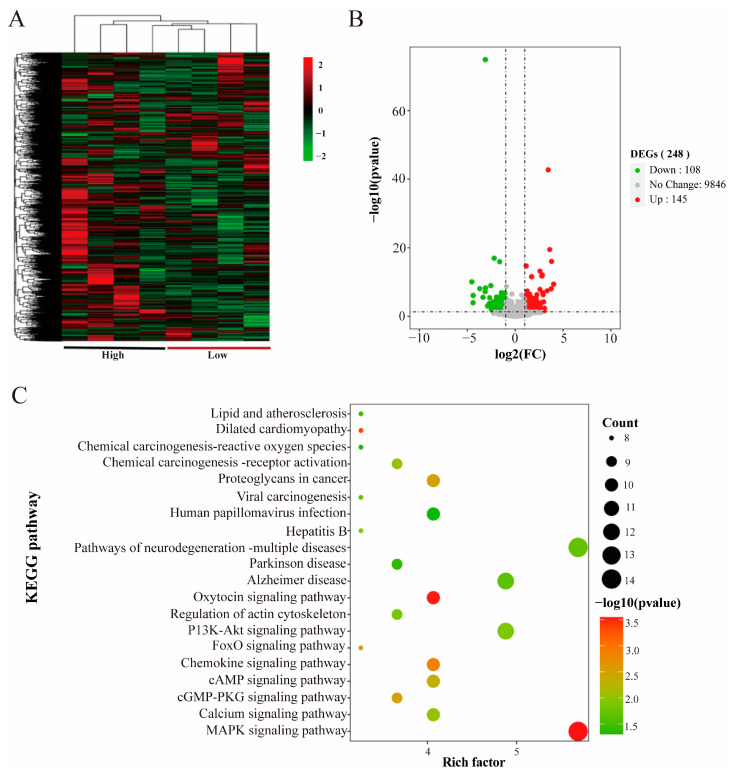
mRNA hierarchical clustering and functional enrichment. (**A**) Heatmap of mRNA hierarchical clustering. (**B**) Volcano map of mRNA differential genes. (**C**) Scatter plot of the top 20 significant KEGG enrichment pathways of mRNA.

Expression analysis by mRNA sequencing was performed on the *longissimus dorsi* samples from the high and low groups. Hierarchical clustering of all samples based on the mRNA expression showed that crossbred pigs with different growth rates exhibited different expression patterns (Figure 2A). The DESeq2 results showed that a total of 248 DEGs were detected in the two groups subjected to differential analyses, of which 145 were upregulated and 103 were downregulated in the high group (Figure 2B). 

GO term and KEGG pathway enrichment analyses were conducted to investigate the biological functions of the differentially expressed genes related to the meat production traits among pigs. The GO term results showed that the DEGs were mainly enriched in biological processes such as skeletal muscle development, cell proliferation, and homeostasis in tissues. The molecular functions were mainly enriched in protein kinase activity, ATP binding, and actin binding. From the results of the CC analysis, the products of differentially expressed genes were mainly distributed in the cytoplasm, cytoskeleton, and muscle membrane (Table S1). The enrichment results of the KEGG pathways were mainly related to muscle growth and development as well as the meat quality and other traits, such as the mitogen-activated protein kinase (MAPK) signaling pathway, the PI3K/AKT signaling pathway, and the calcium signaling pathway (Figure 2C) (Table S2).

Based on the transcriptome sequencing results and the related literature for screening, we identified six mRNAs related to the meat production traits, including *MTMR14*, *PPP1R3A*, *PYGM*, *PGAM2*, *MYH1,* and *MYH7*, as candidate genes.

### 3.4. miRNA Sequencing Expression Analysis

**Figure 3:**
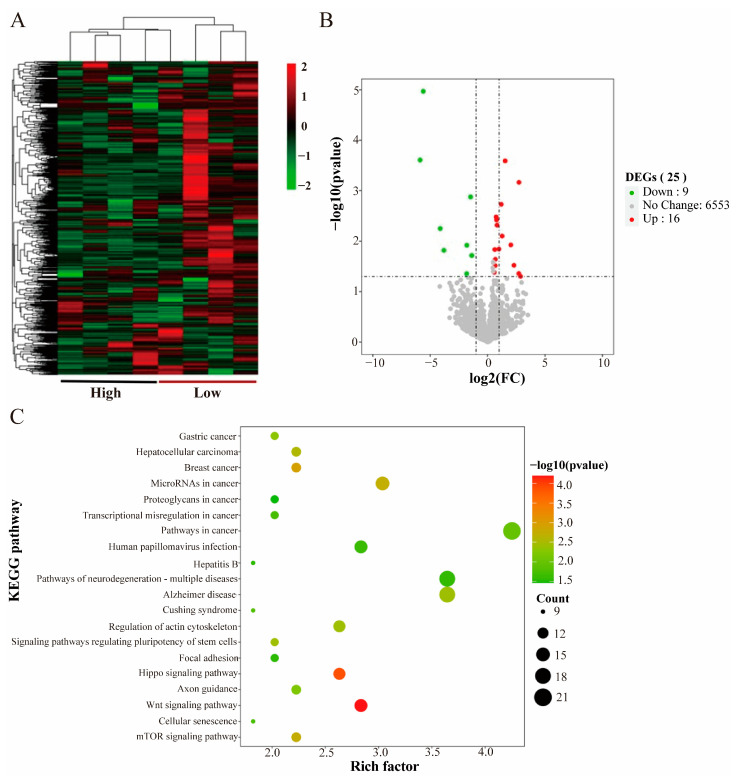
miRNA hierarchical clustering and functional enrichment. (**A**) Heatmap of miRNA hierarchical clustering. (**B**) Volcano map of miRNA differential genes. (**C**) Scatter plot of the top 20 significant KEGG enrichment pathways of miRNA.

Hierarchical clustering of all samples based on the miRNA sequencing expression showed that the high and low groups exhibited different expression patterns (Figure 3A). Statistical analyses of the miRNA expression levels of all samples showed that a total of 25 differentially expressed miRNAs were detected in the high and low groups, of which 16 were upregulated and 9 were downregulated in the high group (Figure 3B).

The target genes of differentially expressed miRNAs, predicted by Targetscan, were enriched. The biological processes were mainly enriched in protein phosphorylation, positive regulation of transcription, and negative regulation of gene expression. Molecular functions were mainly enriched in protein kinase binding, RNA binding, and mRNA binding. The cellular components were enriched mainly in the nucleus, cytoskeleton, and cytoplasm (Table S3). The KEGG-enriched pathways were mainly related to the regulation of sarcomeres and myofibrils, including the Hippo signaling pathway, the mammalian target of rapamycin (mTOR) signaling pathway, and the MAPK signaling pathway (Figure 3C) (Table S4).

Based on the miRNA target genes and enrichment into pathways, we screened two miRNAs associated with the meat production traits, miR-208b and miR-124a-1.

### 3.5. lncRNA Sequencing Expression Analysis

**Figure 4:**
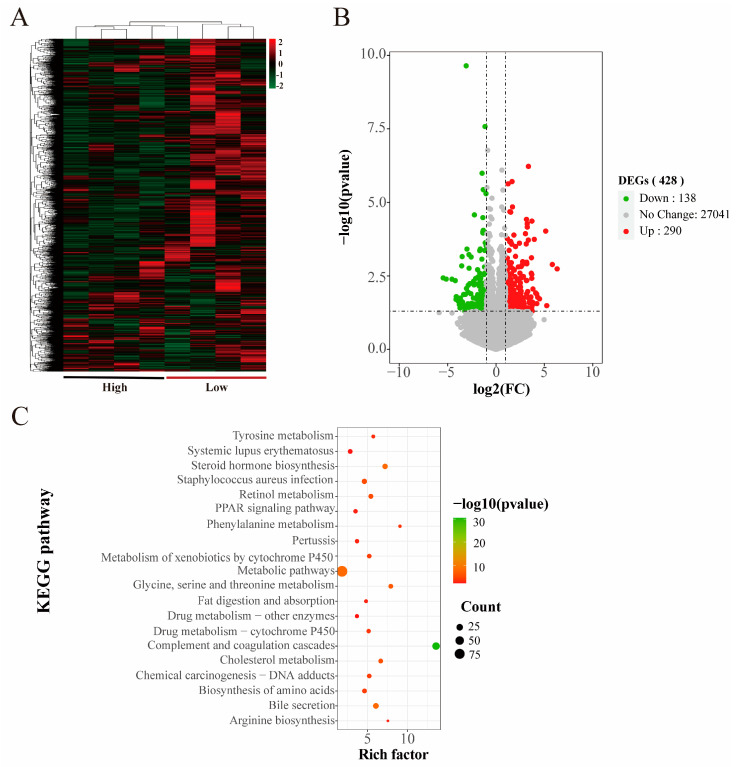
lncRNA hierarchical clustering and functional enrichment. (**A**) Heatmap of lncRNA hierarchical clustering. (**B**) Volcano map of lncRNA differential genes. (**C**) Scatter plot of the top 20 significant KEGG enrichment pathways of lncRNA.

The hierarchical clustering of all samples based on the lncRNA sequencing expression showed that the high and low groups exhibited different expression patterns (Figure 4A). Statistical analyses of the lncRNA expression levels of all samples showed that a total of 432 differentially expressed lncRNAs were detected in the high and low groups, of which 324 were upregulated and 108 downregulated in the high group (Figure 4B). 

GO terms and KEGG enrichment were performed after the target gene prediction of differential lncRNAs. Biological processes were enriched in negative regulation of cytokine activity, cellular responses to lipopolysaccharide, and immune responses. Molecular functions were enriched in phospholipid activity, monooxygenase activity, and oxidoreductase binding. The products of differentially expressed genes were mainly distributed in the nucleus, cytoskeleton, and cytoplasm (Table S5). KEGG was mainly enriched in the PPAR signaling pathway, fatty acid digestion, and uptake, and other pathways related to the meat production traits (Figure 4C) (Table S6).

We analyzed the target genes and enrichment pathways of differential lncRNAs and screened four lncRNAs associated with the meat production traits using the correlation of target genes and pathways with meat production traits, including ENSSSCG00000002475, ENSSSCG00000003349, ENSSSCG00000006245, and ENSSSCG00000006875, and the corresponding target genes were *SERPINA6*, *LRRC38*, *SDR16C5*, and *PLPPR4*, respectively.

### 3.6. ceRNA Regulatory Network Analysis and Quantitative Validation

**Figure 5:**
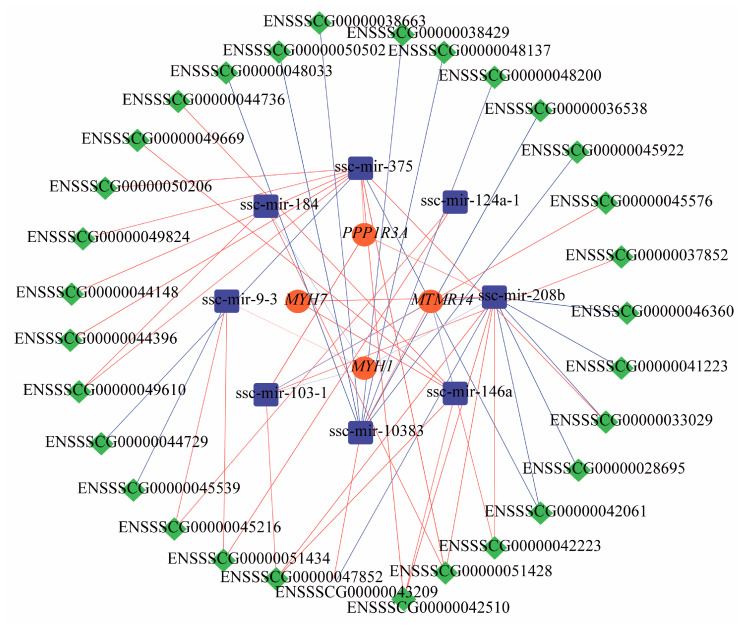
The ceRNA regulatory network related to meat production traits in pigs. Diagram of ceRNA regulatory network. Red represents mRNAs, blue represents miRNAs, and green represents lncRNAs. Red lines represent upregulation, and blue lines represent downregulation.

**Figure 6:**
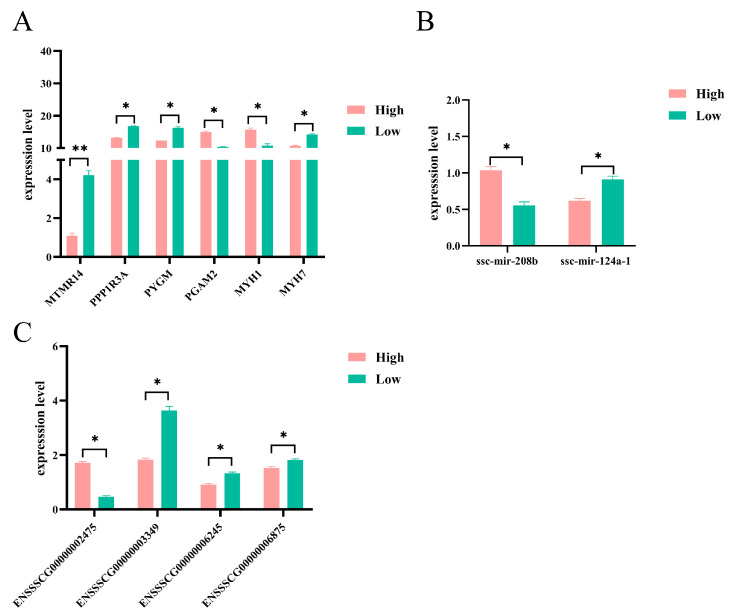
Validation of miRNA, miRNA, and lncRNA expression levels using qRT-PCR. (**A**) Plot of quantitative results of mRNA expression levels. (**B**) Plot of quantitative results of miRNA expression levels. (**C**) Plot of quantitative results of lncRNA expression levels. * Indicates that the results are significantly different (*p* < 0.05). ** Indicates that the results are highly significantly different (*p* < 0.01).

To identify the key lncRNA-miRNA-mRNA interactions associated with meat production, we selected genes with relatively high connectivity and visualized the lncRNA-miRNA-mRNA network using Cytoscape. The screened ceRNA network regulatory pairs associated with the regulation of meat production included ENSSSCG00000042061-ssc-mir-208b-*MYH7*, ENSSSCG00000042223-ssc-mir-146a-*MTMR14*, ENSSSCG00000045539-ssc-mir-9-3-*MYH1*, and ENSSSCG00000047852-ssc-mir-103-1-*PPP1R3A* (Figure 5) (Table S7). Quantitative validation was conducted for the selected genes with relatively high connectivity and relevance to the meat production traits (Table S1). The results were all significantly different between the high and low groups, as expected (*p* < 0.05) (Figure 6A–C) (Table S8).

## 4. Discussion

Differences in the slaughter and meat quality indexes of the F2 generation crossbred pigs were analyzed, and it was found that the loin eye area was significantly higher in the high-growth group than in the low group (*p* < 0.05). The backfat thickness of the thoracolumbar junction was significantly lower in the high group than in the low group (*p* < 0.05). The industry’s single-minded pursuit of pork production has led to a steady decline in meat quality. Under the premise of satisfying the demand quantity, meat quality has become an important factor influencing the consumer’s choice [26]. Research into the meat quality of hybrid pigs is currently a hot topic, owing to its potential for improving lean growth without decreasing the pork’s eating quality [27]. Duroc pigs have high growth rates but a low IMF [28]. The Bama Xiang pig is a unique Chinese fatty pig breed in south China [9]; it has a small body size, excellent meat quality, tolerates roughage, and is more resilient to adversity. These unique advantages make them of great food and scientific value [29]. Skeletal muscles are the main meat-producing tissue of pigs [30]. The results of the statistical analyses of the phenotypic data of the high and low groups illustrate the differences in the phenotypes of crossbred pigs with different growth rates. There was a highly significant difference in the live weight at slaughter in the fast-growing group compared to the slow-growing group. There was also a significant difference in the ocular muscle area between the two groups. Backfat thickness is an important carcass composition trait for pork production [31]. Backfat thickness is related to backfat deposition and is an important indicator of meat quality that is genetically regulated, whereas an increase in adipocyte size is the main reason for an increase in backfat thickness [32]. Our study found that the backfat thickness at the thoracolumbar junction was significantly lower in fast-growing pigs than in slow-growing pigs. The pH value is the most important index of meat quality, determining both the eating quality of the meat and its suitability for processing [33]. It has been demonstrated that higher pH values make pork products more tender, juicy, and flavorful [34,35]. Our measurements illustrate that the pH 24 h of the low group was significantly higher than that of the high group. Intramuscular fat (IMF) is an important indicator of meat sensory qualities, such as share force, juiciness, taste, and nutritional value, which corresponds to the fat deposition between muscle fibers [36,37,38,39]. Increasing the intramuscular fat content of meat is a very important goal in production. The results of this study showed that the intramuscular fat content was significantly higher in the low group than in the high group.

The total protein content in serum is crucial for the health of the organism, and the results of our research revealed that the total protein content of the low group was significantly higher than that of the high group, while the cholesterol content of the low group was significantly higher than that of the high group. High cholesterol intake is a major cause of cardiovascular disease in humans and is the reason why consumers prefer meat products with a low cholesterol content. Numerous studies have shown that soluble fermentable fibers can lead to a reduction in serum cholesterol levels in humans and animals [40]. Although the cholesterol of the slow-growing pigs in this study was higher than that of the fast-growing pigs, it was still within the normal range, which we guessed was due to the higher intramuscular fat content, which was consistent with the previous measurement that the intramuscular fat content of the slow-growing pigs was significantly higher than that of the fast-growing pigs (*p* < 0.05).

The skeletal muscle of pigs is an important source of meat products and an ideal model for muscle growth and development studies [41]. Muscle growth and development are closely related to meat production and are a major factor in the growth of animals [9]. Skeletal muscle fibers are the largest cells in mammals, accounting for 75–90% of the skeletal muscle tissue [42]. The muscle fiber characteristics are closely related to the pork meat’s color, pH, muscle share force, and intramuscular fat (IMF) content [14,43]. The total fiber number and fiber type composition of the skeletal muscle are important aspects of postnatal growth performance, carcass characteristics, and meat quality in pigs, and postnatal muscle development is thought to be caused by myofibril hypertrophy through an increased fiber diameter and length [44]. Our study utilized HE staining of muscle fibers in the *longissimus dorsi* of the porcine dorsum and found that there was no significant difference in the diameters of muscle fibers between the high and low groups. The motor skeletal muscle of adult pigs expresses one slow (MyHC-I) and three fast (MyHC-2a, -2x, and -2b) MyHC isoforms [45]. Fast-twitch muscle fibers (type II) have a greater diameter than slow-twitch (type I) fibers, which is more important in hypertrophy [46]. Different types of muscle fibers differ in their contractile properties and metabolic properties, so the type of muscle fibers that make up a muscle affects the differences in meat production. Huang et al. [47] reported that Bama Xiang pigs have a high longissimus dorsi type I muscle fiber content. Park J et al. demonstrated the predominance of type II fibers over type I fibers in the *longissimus dorsi* of the Duroc [48]. We further determined the typing of myofibers in the *longissimus dorsi* using NADH myocardial xanthanase activity staining subsequently, and found that the proportion of type I myofibers in the low group was significantly higher than that in the high group, while the proportion of type II myofibers was relatively small.

Skeletal muscle growth and development in vertebrates is a complex physiological process, mainly involving the proliferation of myogenic stem cells to form myoblasts and the differentiation and fusion of myoblasts to form multi-nucleated myofibers [49]. RNA-seq is commonly used to study transcriptional regulation during porcine muscle development by comparing different types of breeds, such as lean and fat pigs [50,51,52], and it can also be used in combination with other functional genomics techniques to paint a picture of the mechanisms of muscle development [53]. Whole transcriptome sequencing is a key technology for identifying mRNA and ncRNA and is widely used to elucidate the molecular mechanisms of growth and development, as well as diseases and other related fields [54]. The ceRNA hypothesis has attracted much attention in recent years, and there is growing evidence that lncRNAs may act as ceRNAs for specific miRNAs to regulate miRNA target genes [55]. A total of 248 differentially expressed mRNAs, 25 differentially expressed miRNAs, and 432 differentially expressed lncRNAs were identified in the *longissimus dorsi* of high- and low-group crossbred pigs based on the transcriptome in this study. Functional annotation, target gene prediction, and signaling pathway analysis were performed on these differentially expressed genes.

KEGG is enriched by the multiple pathways associated with meat production and growth and development. Among them, the MAPK signaling pathway can affect the muscle’s structure by altering the expression of related genes in the muscle [56]. MAPK is one of the major regulators of gene transcription in response to oxidative stress in skeletal muscle. It can regulate muscle damage caused by strenuous exercise in skeletal muscle, and the metabolites produced by muscle damage and strenuous exercise can severely affect the meat’s quality [57]. IGF-1 increases skeletal muscle protein synthesis through the PI3K/Akt/mTOR and PI3K/Akt/GSK3β pathways to increase skeletal muscle protein synthesis, and muscle protein renewal leads to reduced muscle mass and strength. PI3K/Akt inhibits FoxO and represses transcription of E3 ubiquitin ligases that regulate ubiquitin-proteasome system (UPS)-mediated protein degradation [58]. The Hippo pathway contributes to myxoma gene expression at the precise timing, thereby generating feedback mechanisms for muscles to precisely coordinate myofibril protein levels during myofibril assembly and maturation, as the Hippo pathway does by regulating myofibril gene expression to control myofibril assembly and myofibril growth [59]. The mTOR pathway is a major regulator of skeletal muscle fiber viability, with the mTOR complexes—mTORC1 and mTORC2—controlling myofiber metabolism, growth, and proliferation [60]. PPARγ is a major regulator of adipogenesis and lipogenesis. PPAR is also expressed in muscle as a major excitatory factor of the PPAR signaling pathway and exerts a pleiotropic specialized response upon ligand activation. PPARα is highly expressed in tissues that efficiently catabolize metabolizable fatty acids, including skeletal muscle. PPARβ/δ is more prevalent in skeletal muscle, and its expression is more prevalent and involved in energy metabolism, mitochondrial biogenesis, and fiber type conversion [61]. Skeletal muscle-specific PPARγ overexpression promotes oxidative fiber formation in pigs [62].

We targeted candidate genes at *MTMR14*, *MYH1*, *MYH7*, *PPP1R3A*, *PGAM2*, and *PYGM,* combining enrichment pathways and previous studies. *MTMR14* plays an important role in regulating muscle performance, autophagy, and senescence in mice, whereas its deficiency induces muscle disorders [63]. MYH1 and MYH7 belong to the MYH family, which has regulatory roles for muscle fibers with regulatory effects. *PPP1R3A* (the gene encoding RGL, a key regulator of muscle glycogen metabolism) was found to be downregulated in chickens when fed methionine, which, in turn, affects the muscle pH [63]. *PYGM* is highly expressed in all tissues of pigs, and a G/T mutation was identified in exon 8, which was significantly associated with lean muscle mass in the correlation analyses [64]. The knockdown of *PYGM* significantly reduces the ability of Lnc-RAM’s function in promoting muscle cell differentiation, and the interaction between Lnc-RAM and *PYGM* was able to positively regulate the enzymatic activity of *PYGM* in muscle cells [65]. *PGAM2* is able to encode key enzymes in glycolysis, and studies have shown that this is a promising marker for improving the water-holding capacity of pork [66].

Some differential miRNAs were also found to be related to muscle fibers. One of these studies verified the targeting relationship between miR-208b and Mettl8 and proved that miR-208b regulates the transformation of different muscle fiber types by inhibiting the expression of Mettl8 [67]. A related genome-wide association study revealing the genomic variants associated with the number and diameter of porcine muscle fibers identified several candidate genes or non-coding RNAs based on their proximity to important SNPs, including miR-124a-1 [68].

We performed target enrichment of lncRNAs and identified several target genes and pathways associated with the meat production traits. The target genes included *SERPINA6*, *LRRC38*, *SDR16C5*, and *PLPPR4*. *SERPINA6* has been associated with obesity and stress sensitivity [69], and excessive obesity in pigs affects the pork’s yield and quality. *PLPPR4* (phospholipid phosphatase-related gene 4) was the target gene screened on Jinghai yellow chickens concerning cell proliferation and differentiation, muscle growth, and cell division. The lncRNAs corresponding to the above target genes are ENSSSCG00000002475, ENSSSCG00000003349, ENSSSCG00000006245, and ENSSSCG00000006875, which may play a role in the regulation of meat production traits. To clarify the regulatory relationship between genes more clearly, we analyzed the mRNAs, miRNAs, and lncRNAs related to the meat production traits by using ceRNA network regulation and found that these genes existed in different ways and that hundreds of ceRNA regulation networks existed. According to the visualization analysis, ENSSSCG00000042061-ssc-mir-208b-*MYH7*, ENSSSCG00000042223-ssc-mir-146a-*MTMR14*, ENSSSCG00000045539-ssc-mir-9-3-*MYH1,* and ENSSSCG00000047852-ssc-mir-103-1-*PPP1R3A* could be the focus of subsequent studies.

## 5. Conclusions

In summary, this study compared the phenotypic traits and biochemical indexes of individuals with different meat production traits in the F2 generation of the Duroc × Bama Xiang pig crossbreed. The whole transcriptome sequencing technology was used to screen the differential genes for meat production traits at the transcriptional level in individuals with different growth rates. Six differentially expressed genes related to meat production, named *MTMR14*, *PPP1R3A*, *PYGM*, *PGAM2*, *MYH1,* and *MYH7*, were focused on. The ceRNA regulatory network maps showed that ENSSSCG00000042061-ssc-mir-208b-*MYH7*, ENSSSCG00000042223-ssc-mir-146a-*MTMR14*, ENSSSCG00000045539-ssc-mir-9-3-*MYH1*, and ENSSSCG00000047852-ssc-mir-103-1-*PPP1R3A* may be the key factors affecting meat production traits through corresponding regulatory relationships. The findings provide a reference for further exploring the mechanism of porcine muscle development and meat quality.

## Figures and Tables

**Table 1:** Analysis of slaughtering indicators and meat quality of Duroc × Bama Xiang F2 hybrid pigs.

Measurement Index		High Group ^1^	Low Group ^1^
Live Weight at Slaughter (kg)		148.38 ± 8.97 ^A^	88.75 ± 9.13 ^B^
Percentage of Skin Fat (%)		40.74 ± 6.02	40.73 ± 2.80
Percentage of Lean Meat (%)		44.05 ± 3.30	43.17 ± 2.81
Dressing Percentage (%)		76.76 ± 1.09	78.08 ± 3.46
Loin Eye Area (cm^2^)		36.13 ± 6.07 ^a^	25.72 ± 4.90 ^b^
Backfat Thickness (mm)	Middle Shoulder	57.83 ± 12.87	58.83 ± 8.73
	Thoracolumbar Junction	29.27 ± 8.12 ^b^	35.58 ± 4.54 ^a^
	Lumbar Sacral Vertebra Junction	31.47 ± 11.07	32.32 ± 2.57
pH 45 min		6.51 ± 0.10	6.40 ± 0.24
pH 24 h		5.69 ± 0.04 ^a^	5.60 ± 0.14 ^b^
Meat Color 45 min		85.43 ± 2.45	84.85 ± 2.55
Meat Color 24 h		65.58 ± 5.30	63.03 ± 6.38
Colorimetric Meat Color Score 45 min		3.50 ± 0.58	3.00 ± 0.00
Colorimetric Meat Color Score 24 h		2.00 ± 0.00	2.00 ± 0.00
Drip Loss 48 h (g)		0.54 ± 0.17	0.48 ± 0.19
Share Force (KGF)		7.82 ± 0.82	7.95 ± 1.52
Moisture (%)		74.17 ± 0.01	73.49 ± 0.01
IMF (%)		2.60 ± 0.008 ^b^	3.99 ± 0.015 ^a^

^1^ Shoulder markers with different lowercase letters indicate significant differences (*p* < 0.05), shoulder markers with different capital letters indicate highly significant differences (*p* < 0.01), and shoulder markers with the same letter indicate non-significant differences (*p* > 0.05).

**Table 2:** Analysis of serum biochemical indices of Duroc × Bama Xiang F2 hybrid pigs.

Plasma Metabolites	High Group ^1^	Low Group ^1^
Total Protein (mg/mL)	31.20 ± 3.00 ^b^	36.23 ± 0.40 ^a^
Cholesterol (mmol/mL)	4.03 ± 0.47 ^b^	5.67 ± 0.92 ^a^
Triglycerides (mmol/mL)	0.86 ± 0.38	1.21 ± 0.19
Creatinine (umol/mL)	630.39 ± 69.83	534.91 ± 72.98
Glucose (mg/mL)	1.82 ± 0.17	1.74 ± 0.33
Urea Nitrogen (mmol/mL)	5.03 ± 1.60	7.40 ± 0.55

^1^ Shoulder markers with different lowercase letters indicate significant differences (*p* < 0.05), and shoulder markers with the same letter indicate non-significant differences (*p* > 0.05).

**Table 3:** Comparison of muscle fiber diameter differences between high and low groups.

Items	High Group ^1^	Low Group ^1^
Muscle Fiber Diameter (μm)	40.87 ± 3.85	40.34 ± 5.68

^1^ Data in the table are not significantly different (*p* > 0.05).

**Table 4:** Comparison of muscle fiber type differences between high and low groups.

Myofiber Type	High Group ^1^	Low Group ^1^
Type I Muscle Fibers (%)	14.1 ± 1.61 ^b^	19.9 ± 1.18 ^a^
Type II Muscle Fibers (%)	85.9 ± 4.77 ^a^	80.1 ± 5.30 ^b^

^1^ Shoulder markers with different lowercase letters indicate significant differences (*p* < 0.05).

## Data Availability

The datasets used and/or analyzed in the current study are available from the corresponding author upon reasonable request.

## Supplementary Materials

The following supporting information can be downloaded at: https://www.mdpi.com/article/10.3390/ani14162347/s1, Table S1: Top 20 GO terms of mRNA. Table S2: Top 20 KEGG enrichment of mRNA. Table S3: Top 20 GO terms of miRNA. Table S4: Top 20 KEGG enrichment of miRNA. Table S5: Top 20 GO terms of lncRNA. Table S6: Top20 KEGG enrichment of lncRNA. Table S7: Log_2_ fold change and *p*-value of the LncRNA-miRNA-mRNA network. Table S8: Quantitative validation primer sequences.
