# Supplementary material for: Screening for Genes Related to Meat Production Traits in Duroc × Bama Xiang Crossbred Pigs by Whole Transcriptome Sequencing
Source: Animals (Basel). 2024 Aug 14;14(16):2347. doi: 10.3390/ani14162347 (PMC11350711; doi:10.3390/ani14162347)
Supplement: Supplementary file 1 [file animals-14-02347-s001.zip › animals-3108232-supplementary.pdf]

Table S1. Top20 GO Terms of mRNA

ID	Term	Count	*P*-value	*P-a*djust
GO:1990868	response to chemokine	5	2.03E-05	0.015910944
GO:1990869	cellular response to chemokine	5	2.03E-05	0.017618024
GO:0016529	sarcoplasmic reticulum	4	2.37E-05	0.003905758
GO:0016528	sarcoplasm	4	5.46E-05	0.004507213
GO:2000403	positive regulation of lymphocyte migration	4	5.71E-05	0.029838541
GO:1903169	regulation of calcium ion transmembrane transport	5	0.000127587	0.032142927
GO:0090257	regulation of muscle system process	6	0.000142042	0.0409819
GO:0097529	myeloid leukocyte migration	6	0.000171809	0.0409819
GO:0051279	regulation of release of sequestered calcium ion into cytosol	4	0.000177964	0.052226559
GO:0042692	muscle cell differentiation	8	0.000208531	0.052837162
GO:0070098	chemokine-mediated signaling pathway	4	0.000215201	0.051634138
GO:2000401	regulation of lymphocyte migration	4	0.000215201	0.057327165
GO:0070588	calcium ion transmembrane transport	6	0.000225492	0.076582138
GO:0043500	muscle adaptation	4	0.00025774	0.033678091
GO:0050848	regulation of calcium-mediated signaling	4	0.000306005	0.035473978
GO:0051924	regulation of calcium ion transport	6	0.000316732	0.023473978
GO:0070296	sarcoplasmic reticulum calcium ion transport	3	0.000401691	0.036209656
GO:1901623	regulation of lymphocyte chemotaxis	3	0.000401691	0.036206756
GO:0060401	cytosolic calcium ion transport	5	0.000415672	0.034309656
GO:0097530	granulocyte migration	5	0.000415672	0.036209655

Table S2. Top20 KEGG Enrichment of mRNA

ID	Pathway	Count	*P*-value	*P-a*djust
ssc05417	Lipid and atherosclerosis	8	0.014544972	0.091633323
ssc05414	Dilated cardiomyopathy (DCM)	8	7.31E-05	0.004228028
ssc05208	Chemical carcinogenesis-reactive oxygen species	8	0.018590858	0.097231575
ssc05207	Chemical carcinogenesis -receptor activation	9	2.88E-03	0.034553639
ssc05205	Proteoglycans in cancer	10	0.000886852	0.015963342
ssc05203	Viral carcinogenesis	8	0.007269409	0.05909326
ssc05165	Human papillomavirus infection	10	0.022483057	0.106454698
ssc05161	Hepatitis B	8	0.00315559	0.034574291
ssc05022	Pathways of neurodegeneration -multiple diseases	14	9.66E-03	0.068988023
ssc05012	Parkinson disease	9	0.017115642	0.097231575
ssc05010	Alzheimer disease	12	0.010757191	0.071337159
ssc04921	Oxytocin signaling pathway	10	6.10E-05	0.004228028
ssc04810	Regulation of actin cytoskeleton	9	0.006356806	0.053397173
ssc04151	PI3K-Akt signaling pathway	12	0.004952717	0.04622536
ssc04068	FoxO signaling pathway	8	6.36E-04	0.013360169
ssc04062	Chemokine signaling pathway	10	3.36E-04	0.008628274
ssc04024	cAMP signaling pathway	11	4.61E-04	0.010572052
ssc04022	cGMP-PKG signaling pathway	9	7.69E-04	0.014898859
ssc04020	Calcium signaling pathway	10	3.12E-03	0.034574291
ssc04010	MAPK signaling pathway	14	8.39E-05	0.004228028

Table S3. Top20 GO Terms of miRNA

ID	Term	Count	*P*-value	*P-a*djust
GO:0006355	regulation of transcription, DNA-templated	31	0.019054607	0.003905758
GO:0006357	regulation of transcription from RNA polymerase II promoter	62	2.51E-11	0.004507563
GO:2000144	positive regulation of transcription, DNA-templated	21	0.002754229	0.029838541
GO:0060261	positive regulation of transcription from RNA polymerase II promoter	39	2.69E-06	0.057650303
GO:0010628	positive regulation of gene expression	19	0.021379621	0.023473238
GO:0018107	peptidyl-threonine phosphorylation	9	2.09E-05	0.043616559
GO:0060633	negative regulation of transcription from RNA polymerase II promoter	38	3.31E-08	0.052837162
GO:1990830	cellular response to leukemia inhibitory factor	11	0.00020893	0.051634138
GO:0030154	cell differentiation	19	0.025703958	0.057327165
GO:0009952	anterior/posterior pattern specification	12	0.000213796	0.076582138
GO:0001228	transcriptional activator activity, RNA polymerase II transcription regulatory region sequence-specific binding	40	1.15E-09	0.065022883
GO:0003700	transcription factor activity, sequence-specific DNA binding	36	2.45E-08	0.021211642
GO:0000981	RNA polymerase II transcription factor activity, sequence-specific DNA binding	59	3.16E-11	0.03673098
GO:0044377	RNA polymerase II core promoter proximal region sequenca-specific DNA binding	70	6.31E-14	0.020547651
GO:0008023	transcription factor complex	32	2.75E-07	0.066487914
GO:1903353	nucleus	148	0.001778279	0.078760109
GO:0005654	nucleoplasm	98	2.24E-05	0.066487914
GO:0099522	cytosol	146	1.10E-09	0.072455309
GO:0099568	cytoplasm	149	2.14E-06	0.036209656
GO:0006355	regulation of transcription, DNA-templated	31	0.019054607	0.003905758

Table S4. Top20 KEGG Enrichment of miRNA

ID	Pathway	Count	*P*-value	*P-a*djust
ssc05226	Gastric cancer	10	0.005623413	0.047175056
ssc05225	Hepatocellular carcinoma	11	0.002137962	0.008628274
ssc05224	Breast cancer	11	0.001230269	0.025142892
ssc05206	MicroRNAs in cancer	15	0.00128825	0.004228028
ssc05205	Proteoglycans in cancer	10	0.026915348	0.029006139
ssc05202	Transcriptional misregulation in cancer	10	0.020417379	0.059137237
ssc05200	Pathways in cancer	21	0.019498446	0.025211548
ssc05165	Human papillomavirus infection	15	0.026915348	0.105043935
ssc05161	Hepatitis B	9	0.018620871	0.106454698
ssc05022	Pathways of neurodegeneration - multiple diseases	18	0.024547089	0.068988023
ssc05010	Alzheimer disease	18	0.002089296	0.017404928
ssc04934	Cushing syndrome	9	0.004265795	0.175347045
ssc04810	Regulation of actin cytoskeleton	13	0.002754229	0.111314974
ssc04550	Signaling pathways regulating pluripotency of stem cells	10	0.003090295	0.097231575
ssc04510	Focal adhesion	10	0.024547089	0.05934973

ssc04390	Hippo signaling pathway	12	0.00020893	0.072865118
ssc04360	Axon guidance	11	0.004265795	0.019006139
ssc04310	Wnt signaling pathway	14	8.51E-05	0.059137237
ssc04218	Cellular senescence	9	0.020892961	0.124036069
ssc04150	mTOR signaling pathway	11	0.001380384	0.053397173

Table S5. Top20 GO Terms of lncRNA

ID	Term	Count	*P*-value	*P-a*djust
GO:1903034	regulation of response to wounding	26	7.94E-37	0.0309819
GO:002639	positive regulation of immunoglobulin production	27	6.31E-35	0.0402819
GO:0002904	positive regulation of cytokine production	27	3.16E-28	0.052226559
GO:0001819	positive regulation of B cell apoptotic process	26	2.51E-39	0.052837162
GO:1904706	negative regulation of vascular smooth muscle cell proliferation	27	5.01E-33	0.035473978
GO:0032720	negative regulation of tumor necrosis factor production	27	2.00E-26	0.023473978
GO:0030886	negative regulation of myeloid dendritic cell activation	26	3.98E-36	0.057327138
GO:0032715	negative regulation of interleukin-6 production	27	3.16E-28	0.066487914
GO:0032695	negative regulation of interleukin-12 production	27	1.26E-34	0.073745309
GO:0032649	negative regulation of interferon-gamma production	28	1.58E-30	0.080192705
GO:1903208	negative regulation of hydrogen peroxide-induced neuron death	24	2.51E-38	0.086042705
GO:2000352	negative regulation of endothelial cell apoptotic process	28	2.00E-29	0.089092705
GO:0060302	negative regulation of cytokine activity	24	7.94E-38	0.090862526
GO:0002875	negative regulation of chronic inflammatory response to antigenic stimulus	23	1.58E-36	0.084143657
GO:0010507	negative regulation of autophagy	27	2.51E-30	0.106767154
GO:0042832	defense response to protozoan	27	3.16E-18	0.106017599
GO:0030170	pyridoxal phosphate binding	10	3.31E-08	0.103460599
GO:0005125	cytokine activity	7	1.74E-05	0.108903036
GO:0005615	extracellular space	98	2.51E-39	0.085014705
GO:0005576	extracellular region	76	3.98E-26	0.073105605

Table S6. Top20 KEGG Enrichment of lncRNA

ID	Pathway	Count	*P*-value	*P-a*djust
ssc00350	Tyrosine metabolism	5	0.000338844	0.015910944
ssc05322	Systemic lupus erythematosus	7	7.76E-05	0.003940226
ssc00140	Steroid hormone biosynthesis	11	3.02E-09	0.003905758
ssc05150	Staphylococcus aureus infection	11	2.04E-08	0.004507213
ssc00830	Retinol metabolism	10	1.38E-07	0.029838541
ssc03320	PPAR signaling pathway	5	0.002041738	0.056849303
ssc00360	Phenylalanine metabolism	4	2.09E-05	0.017587677
ssc05133	Pertussis	7	0.002630268	0.063942863
ssc00980	Metabolism of xenobiotics by cytochrome P450	8	0.003548134	0.065022883
ssc01100	Metabolic pathways	74	2.51E-13	0.021211642

ssc00260	Glycine, serine and threonine metabolism	7	5.50E-10	0.03673098
ssc048975	Fat digestion and absorption	5	0.00020893	0.044117064
ssc00983	Drug metabolism – other enzymes	5	0.003235937	0.097231575
ssc00982	Drug metabolism – cytochrome P450	7	0.000218776	0.033678091
ssc04160	Complement and coagulation cascades	7	2.51E-27	0.06982651
ssc04979	Cholesterol metabolism	7	2.51E-11	0.035473978
ssc05204	Chemical carcinogenesis – DNA adducts	8	2.04E-09	0.006454698
ssc01230	Biosynthesis of amino acids	6	2.19E-09	0.068988023
ssc04976	Bile secretion	15	2.51E-13	0.017404928
ssc00220	Arginine biosynthesis	3	0.003467369	0.040395651

Table S7. Log_2 FoldChange and *P*-value of the LncRNA-miRNA-mRNA Network

Genename	Log_2 FoldChange	*P*-value
MYH7	-1.078698068	0.010871509
MTMR14	-1.621066925	1.2E-16
MYH1	1.068396206	0.019949684
PPP1R3A	-1.011619781	0.042061786
ssc-mir-208b	1.532694548	0.000256174
ssc-mir-146a	1.578765195	0.041590949
ssc-mir-9-3	-1.805313145	0.01204367
ssc-mir-103-1	1.660223689	0.030532889
ENSSSCG00000042061	-1.093095953	0.007237975
ENSSSCG00000042061	-1.085294037	0.009313791
ENSSSCG00000045539	2.262212297	0.012323984
ENSSSCG00000047852	-1.039282623	0.049431297

Table S8. Quantitative Validation Primer Sequences

Genename	Forward Primer (5'→3')	Reverse Primer (5'→3')
MYH7	GGTATCGCATCCTGAACCC	GCCCTGCCTTGAAGAACAC
MTMR14	CTTGGCAGCGACTTCTCTCT	CTCAGGGCTGCAAGCCAA
MYH1	GGCAAGCAAGCATTCACACA	GGCACTCTTGGCCTTGATCT
PPP1R3A	AGCCATCGCTGGGAAATACC	TCCCTTGGGCTGGATTAGCA
PYGM	AAGAACTTCAACCGCCACCT	TGTGGGCCAGTGCAAAGTAG
PGAM2	CCAGATCAAGGCAGGCAAGA	GGCAGGTTCAGCTCCATGAT
ssc-mir-208b	GGAAGCTTTTTGCTCGCGTTA	CTCTGCCCTCAGACAAACCTT
ssc-mir-146a	CTTTGAGAACTGAATTCCATGGGT	CTTTGAGAACTGAATTCCATGGGT
ENSSSCG00000002475	ATCTTTGCTTCAGCCCTGGTC	ATTAACGTGGGAGCGGATGA
ENSSSCG00000003349	AGCTTCAGCGAGTGCAAGTT	GGCCAGAAAGAAGCTGGAGAT
ENSSSCG00000006245	CCTTGAGTGGGCCCATTTGT	CCGGGTGGCAGTTCTATACC
ENSSSCG00000006875	CCACTCCTCTCTGGTCTTGGA	GCATGGCATGTGACAGAGAGA
